# Influence of Expander Conditioning Prior to Pelleting on Pellet Quality, Broiler Digestibility and Performance at Constant Amino Acids Composition while Decreasing AME_N_

**DOI:** 10.3390/ani12223126

**Published:** 2022-11-13

**Authors:** Marco Antônio Ebbing, Nadia Yacoubi, Victor Naranjo, Werner Sitzmann, Martin Gierus

**Affiliations:** 1 Department of Agrobiotechnology (IFA-Tulln), Institute of Animal Nutrition, Livestock Products and Nutrition Physiology (TTE), University of Natural Resources and Life Sciences, 1190 Vienna, Austria; 2 Evonik Operations GmbH, Rodenbacher Chaussee 4, D-63457 Hanau, Germany; 3 Evonik Guatemala S.A., 18 Calle 24-69, Edificio Zona Pradera, Torre 4, Oficina 810, Guatemala City 01010, Guatemala; 4 Amandus Kahl GmbH & Co. KG, Dieselstrasse 5, D-21465 Reinbek, Germany

**Keywords:** feed processing, feed technology, high-temperature short-time conditioning, serum, corn, soybean meal

## Abstract

**Simple Summary:**

Modern broilers convert nutrients and energy into meat very well, responding especially well to dietary amino acids. Currently, their ability for rapid growth allows them to achieve their target weight sooner. This is partly related to their positive response to dense amino acids relative to apparent metabolizable energy corrected for Nitrogen (AME_N_) diets; as recently shown in research, broilers’ energy requirement is lower than that recommended by the strain’s guidelines. On the other hand, feed intake is the key factor that allows the conversion of nutrients into increased body weight. Thus, this trial proposed the investigation of diets with constant amino acid composition with a stepwise AME_N_ reduction, conditioned with or without an expander prior to pelleting, aiming to produce high-quality pellets. The hypothesis was that the non-nutritional factor expander may replace the dietary reduction in AME_N_ without issues on performance, enhancing broiler nutrients digestibility, and maintaining the birds’ health. The results showed that broilers’ performance was only slightly affected by the energy reduction and the use of expanders in the overall period. However, the use of expanders was advantageous at growing and finisher diets, probably due to the disruption of the corn oil cells, allowing faster attachment of endogenous lipase, which is extremely low in the first days and well established after 14 days of age. Broilers responded to the better pellets and higher nutrient digestibility promoted by the expander. In contrast, the AME_N_ reduction allowed lower abdominal fat accumulation, and no issues in broiler health were attested by serum markers.

**Abstract:**

Physical pellet quality and AME_N_ concentration are strongly related to each other in broiler feeding. A study was conducted to evaluate the relationship between dietary AME_N_ concentration and feed processing on pellet quality, nutrient digestibility, broiler performance, serum markers, and yield of commercial cuts. Six diets were formulated. The first diet had the recommended AME_N_ concentration, each further diet was calculated with 40 kcal/kg less, from 0 to −200 kcal/kg, resulting in six levels for each feed phase: starter (1–14 d), grower (15–28 d), and finisher (29–35 d). These diets were processed with and without expander conditioning prior to pelleting, using an average corn particle size of 1.6 mm, ground with a roller mill. A total of 1008 one-day-old male Ross 308 broiler chickens were placed in a 6 × 2 (6 energy levels and 2 conditionings) factorial trial with six boxes as replications, with three in each broiler performance trial period. Excreta were collected 2 days before the end of each feed phase for apparent total tract digestibility measurement. On day 36, four broilers from each replication (pen) were weighed and then euthanized for blood collection, following which the gastrointestinal organs were weighed, and the ileal and gizzard contents were collected. On day 37, all remaining broilers were slaughtered after fasting to measure commercial cuts and abdominal fat. The results show that the pellet durability index (PDI) was most affected by energy reducing and expander conditioning prior to pelleting, and it was better when diets had energy reduced by 40 to 200 kcal/kg (*p* > 0.001), as when expander conditioning was used. Digestibility of nutrients was slightly affected by treatments, as was the broiler performance; however, feed efficiency was improved in broiler-fed diets without AME_N_ reduction and when an expander was used, with *p* = 0.050 and *p* = 0.031, respectively. No effects were observed on the weight of gastrointestinal tract organs and serum markers, except for liver (*p* = 0.037) and α-amylase (*p* = 0.047). The lowest liver weight and lowest serum protein, cholesterol, triglyceride, gamma-glutamyl, and lipase concentrations were obtained when diets were formulated without energy reduction (Ross-0). There was no effect on commercial cuts relative to live weight at slaughter. The energy reduction was well reflected in the proportion of abdominal fat, which decreased when AME_N_ was reduced (*p* = 0.001). The present study shows it is possible to use diets with up to 200 kcal/kg reduction in AME_N_ without losses in performance, and the use of expander conditioning prior to pelleting promotes higher pellet quality and broiler feed efficiency.

## 1. Introduction

Energy-rich ingredients are expensive in broiler diets [1,2]. Maximizing the retention of energy for growth by applying a non-nutritive factor should therefore be considered. This can be achieved by enhancing the physical quality of pellets, or perhaps by increasing digestibility through feed processing, for instance by using the expander for compound feed [3]. However, diets should achieve the broilers’ requirements for nutrients and energy during each growth phase, and it is necessary to consider that the expander conditioning achieved by applying steam, water, and pressure on the compound feed, affects the digestibility of nutrients and energy [4,5]. In this regard, the influence of feed processing should not be overlooked during feed formulation. However, estimating the contribution of feed processing for higher energy values of compound feed before diet formulation is still unclear. In addition to aspects of AME_N_ concentration, recent studies suggest that the energy content in compound feed for broilers may be reduced (lower than recommended by breeding lines) [6,7]. In fact, modern fast-growing broilers achieve the required slaughter weight over a shorter period. However, slaughtering the broilers at the correct time is important, and it is advantageous for it to be undertaken when the growth rate of the muscles is high in relation to the energy required for maintenance. After that, the requirements shift, with high energy required for a lower growth rate.

In the processing of compound feed for broilers, conditioning prior to pelleting is one of the most important steps in the processing line. The high-temperature short-time (HTST) technology with expanders, which apply heat, achieving temperatures above 110 °C for a short time, pressure, and shear forces to the compound feed, has the potential to modify the digestibility of fat [8], amino acids, and starch [9,10]. Non-nutritive factors of compound feed offered to broilers, such as the pellet durability index (PDI) and low proportion of fines, have important contributions affecting feed intake, digestibility, and finally, the amount of energy retention by broilers [3]. Higher energy retention is achieved by reducing the eating time. High pellet quality allows a high amount of feed apprehension per head movement. This contribution should already be considered during feed formulation, but, as mentioned above, the contribution that feed processing has on improvements in nutritive value and animal response on the increase in AME_N_ concentration has been poorly quantified until now. In fact, most broilers worldwide are fed with pelleted diets [4], where modifications on the nutritive value of processed diets already occur. Therefore, the improvement of pellet quality using compound feed processing parameters should investigate the contribution of the different processing steps on the nutritive value of pelleted compound feed for broilers.

The objective of this study was to measure the effects of gradual AME_N_ reduction and expander conditioning prior to pelleting on nutrient digestibility, AME_N_ concentration in each feed phase, broiler performance, and blood serum markers. The main aim was to determine the optimal broiler-diet AME_N_ concentration promoted by expander conditioning prior to pelleting, while keeping the amino acid content constant (variable AME_N_ to amino acid ratio). We hypothesized that AME_N_ reduction decreases broiler performance and that using expander conditioning prior to pelleting may minimize this decrease, without negative influences on metabolic indices.

## 2. Materials and Methods

### 2.1. Diets

Corn-SBM-based broiler diets were formulated using previously analyzed amino acid content of corn (*Zea mays*) and SBM (AMINONir^®^ Advanced, Evonik Operations GmbH, Hanau, Germany) for 6 treatments. The estimated digestibility coefficients of AA and AME_N_ concentration for broilers of corn and SBM was used for the calculation of the diet using digestible nutrients as a basis, following the AMINOChick^®^ recommendations [11]. The ingredients composition of starter, grower, and finisher diets of calculated and analyzed nutrients are shown in Table 1, Table 2 and Table 3, respectively. Nutritive value of these diets aimed to follow or exceed the lineage recommendations [12]. The AME_N_ content in diets followed the requirements for the treatment Ross-0. For the other 5 diets, there was a 40 kcal/kg decrease performed step-by-step to reduce dietary AME_N_ for the starter, grower, and finisher feed phases (mainly by reducing the soybean oil). Each diet had the same and constant digestible AA content and the relationship between them as used in the Ross-0 treatment. This means that the formulation of feeds only decreased the AME_N_ concentration, changing the Lys:AME_N_ ratio among treatments.

### 2.2. Feed Processing Design

The above diets were submitted to two kinds of compound feed processing, resulting in a 6 × 2 factorial design with six AME_N_ levels and two expander conditionings prior to pelleting (OE 15 expander, Amandus Kahl, Reinbek, Germany). The corn was ground with a roller mill (LWM 400-1, Amandus Kahl, Reinbeck, Germany) to obtain 1.6 mm average particle size for all diets, which were mixed for 5 min in a ploughshare mixer (Lödige FKM 1200 D, Amandus Kahl, Reinbek, Germany). Pellets were produced using a 3 mm diameter hole die, installed in a pellet press (Type 33-390, Amandus Kahl, Reinbek, Germany), with 1:4 press ratio (3 mm diameter × 12 mm hole length). Starter, grower, and finisher diets were used as replicates for the feed processing step of the trial, considering each phase (diet) a block in the statistical model (Equation (4)). The starter diet was fed as crumbled, as was the grower diet from 15 to 21 days of age. From day 22 to the end of the trial the broilers received pellet diets with 3 mm of diameter and ~0.8 cm of length.

### 2.3. Pellet Quality Measurements

Pellet durability index (PDI) was measured on cooled pellets by the P-fost method [13] and pellet hardness with the automatic pellet hardness tester (Amandus Kahl, Reinbek, Germany). Briefly, 10 randomly selected pellets from each replication were submitted to a force (kg/cm^2^) until the first fracture. This force was recorded, and the mean value was used for statistical analysis.

### 2.4. Broiler Experimental Design and Housing

A total of 1008 one-day-old male Ross 308 broiler chickens vaccinated against Marek were placed in 36 pens (5.5 birds/m^2^) littered with wood shavings and equipped with one bell drinker and one tubular feeder. Birds had *ad libitum* access to water and feed. The environmental temperature was controlled automatically using infrared lamps (one per box) as a heat source and exhaust fans to supply ventilation and cooling.

The temperature of 32 °C used in the feeding trial follows the strain recommendations, reducing by one degree every two days until achieving 24 °C. The light regime was 23 h dark and 1 h light until 7 days of age and then 7 h dark and 17 h light afterward.

### 2.5. Performance Data

Body weight (BW) and feed intake (FI) were recorded at the end of each feed phase, by weighing the birds without fasting and the non-consumed feed. BWG and FCR were calculated for each phase and overall period. FCR was adjusted by including the weight of dead birds.

### 2.6. Determination of Apparent Total Tract Digestibility and Metabolized Energy

Titanium dioxide (TiO_2_) was used as an indigestible marker. Partial excreta samples were taken, placing all the birds of each pen in metal cages with steel plates for two h at the end of each feed phase. Excreta were packed into plastic bags prior to being stored in freezer at −20 °C for further analyses.

### 2.7. Determination of the Coefficients of Apparent Ileal Digestibility, pH of Gizzard Content, and Blood Sampling

On day 36 of age, 4 birds with average weight ± 2.5% from each pen were taken, stunned by percussive blow to the head, then bled through the jugular vein cut for 3 min. Blood was sampled at the bled moment in tubes with coagulation activator, then centrifuged and stored in cooled boxes and sent to an external laboratory. The gastrointestinal tract was removed, and the gizzard opened for pH measurement of the gizzard content, then weighed empty. The weight of the pancreas and liver were also determined and expressed as relative values to the body weight. Digesta samples were taken from the ileum between Merkel diverticulum and 2 cm cranial to the ileo-cecal junction by flushing with distilled water, packed in plastic bags, and stored in a freezer at −20 °C for further analyses. Prior to analyses, samples were freeze-dried.

### 2.8. Slaughter and Commercial Cuts

On day 37, all remaining broilers were fasted for 8 h, individually weighed, stunned by percussive blow to the head, then bled through a jugular vein cut for 3 min, scalded at 60 °C for 45 s, and defeathered. Evisceration was performed manually, and carcasses were statically chilled in the cooling room at 4 °C for 24 h. Commercial cuts were performed by a crew of industry-trained personnel into bone-in legs, wings, and also as deboned breast fillets with tenders. Abdominal fat was weighed separately. Carcass yield was expressed relative to the live weight, while commercial cuts and abdominal fat were expressed relative to eviscerated carcass weight.

### 2.9. Chemical and Blood Analyses

All diets were analyzed for dry matter (DM) (method 3.1.4), ash (method no. 8.1.1), ether extract after acid hydrolysis (EEh) (method 5.1.2), starch (method 7.2.1) according to standard procedures of VDLUFA [14]. Total N was determined by Dumas’ method (method 968.06) [15] (Büchi, DuMaster D-480, Flawil, Switzerland) and the result multiplied by 6.25 to obtain crude protein. Gross energy concentration was determined by a calorimeter calibrated with benzoic acid as a standard (IKA C 200, Parr instruments, Staufen, Germany). Amino acids content was determined by ion-exchange chromatography with post-column derivatization with ninhydrin, as described by Figueiredo-Silva et al. [16]. Wet-ashing in a microwave oven (CEM Mars 6, CEM Corp. Matthews, NC, USA) was applied to analyze Ca by flame atomic absorption spectrophotometry (AAnalyst 200, Perkin Elmer Inc., Waltham, MA, USA), and P photometrically (Tecan Group Ltd., Männedorf, Switzerland) using the vanado-molybdate method at 436 nm. TiO_2_ content in feed, digesta, and excreta was analyzed following Jagger et al. [17]. In ileal content, DM, AA, starch, and TiO_2_ were analyzed. However, in excreta, only DM, EEh, gross energy, N, ash, and TiO_2_ were analyzed. From blood serum, total cholesterol (method: CHOD-PAP), triglycerides (method: GPO-PAP), lipase (method: enzymatic and colorimetric), gamma-glutamyl transferase (GGT) (method: IFCC), alpha-amylase (method: modified IFCC), and total protein (method: Biuret) were analyzed.

### 2.10. Calculations

Total tract digestibility and AME_N_ were calculated using the equations suggested by Kong and Adeola [18]:

Digestibility (%) = [1 − (M_d_/M_e_) × (E_d_/E_e_)] × 100, (1)

AME_N_ (kcal/kg) = GE_d_ − [GE_e_ × (M_d_/M_e_)] − 8.22 × {N_d_ − [N_e_ × (M_d_/M_e_)]}, (2)

where M_d_ represents the concentration of TiO_2_ in the diet in g/kg; M_e_ represents the concentration of TiO_2_ in the excreta and ileal digesta in g/kg; E_d_ represents the content of total fat, starch, amino acids in g/kg, and gross energy (kcal/kg) in the diet. E_e_ represents the amount of total fat (g/kg) in excreta; and starch and amino acids (g/kg) in ileal digesta. GE_d_ represents the gross energy of diet (kcal/kg), and GE_e_ represents the gross energy of excreta (kcal/kg). N_d_ represents nitrogen (g/kg) in diet and N_e_ represents nitrogen (g/kg) in excreta. All the nutrients and energy were used in DM basis for the calculations.

### 2.11. Statistical Analyses

The experiment design was a completely randomized factorial arrangement of 6 AME_N_ levels and 2 expander conditionings prior to pelleting (with and without expander). All data were subjected to a normality test using Shapiro–Wilk test [19] prior to the 2-way ANOVA using the GLM procedures from SAS Institute [20]. When significant, means were separated by Tukey–Kramer [21] and accepted as different when *p* < 0.05.

The model used was:

Y_ijk_ = µ + γ_i_ + α_j_ + β_k_ + (αβ)_jk_ + ε_ijk_ (3)

where Y_ijk_ = observation, µ = population mean, γ_i_ = broiler performance period effect (i = 1, 2), α_j_ = AME_N_ concentration effect (j = Ross-0, Ross-40, Ross-80, Ross-120, Ross-160, Ross-200), β_k_ = expander effect (k = with, without) and (αβ)_jk_ = interaction between AME_N_ concentration and expander effect, and ε_ijk_ = residual error.

Statistical analysis for feed processing parameters followed the following model:

Y_ijk_ = µ + γ_i_ + α_j_ + β_k_ + (αβ)_jk_ + ε_ijk_ (4)

where Y_ijk_ = observation, µ = population mean, γ_i_ = block effect (i = starter, grower, finisher), α_j_ = AME_N_ concentration effect (i = Ross-0, Ross-40, Ross-80, Ross-120, Ross-160, Ross-200), β_j_ = expander effect (j = with, without), and (αβ)_ij_ = interaction between AME_N_ concentration and expander effect, and ε_ijk_ = residual error. Compound feed for starter, grower, and finisher was used as blocks (r = 3).

## 3. Results

The main feed processing parameters are shown in Table 4. No effect of the interaction AME_N_ concentration vs. expander conditioning prior to pelleting was observed, except for PDI. The statistical analyses for main effects showed a clear influence of reduction in AME_N_ concentration on the pressure (*p* < 0.001) needed in the pellet press to achieve a similar output of 2 t/h. The specific mechanical energy (SME) used by the pellet press increased (*p* < 0.001) when AME_N_ concentration was reduced in the diet. Reducing AME_N_ concentration in diets increased pellet hardness (*p* < 0.001) without significantly changing the proportion of fines (Table 4).

In contrast, expander conditioning prior to pelleting affected diet processing. Using expander conditioning prior to pelleting showed clear differences: more steam was added to the compound feed prior to expanding. In addition, lower SME input (kWh/t) was necessary in the pellet press when expander conditioning was used prior to pelleting. The proportion of fines is positively affected (reduced) by expanding prior to pelleting (*p* < 0.001).

Table 5 shows the interaction effects for the PDI values. The PDI values of the compound feed were, in general, lower when expander conditioning prior to pelleting was not used. For treatments Ross-0 and Ross-40, expander conditioning prior to pelleting could significantly increase the PDI value.

**Figure 1:**
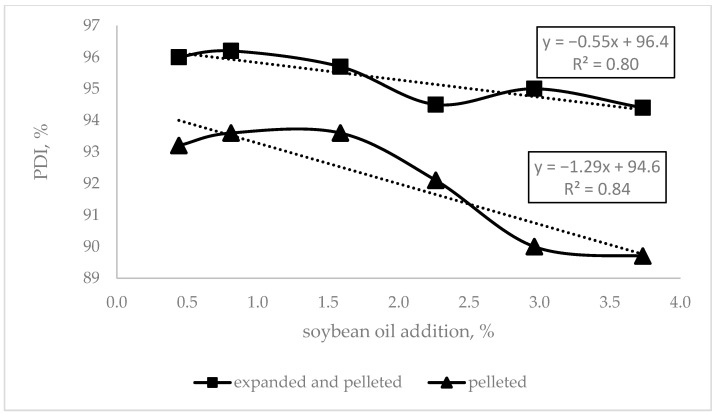
Oil addition (%) effect on pellet durability index (PDI) of pelleted diets conditioned with or without expander prior to pelleting.

Evaluating the effect of the diets’ oil inclusion, Figure 1 shows that the higher the oil content, the poorer the pellets.

The apparent total tract digestibility (ATTD) and AME_N_ concentration measured at the end of each feed phase are shown in Table 6. The effects detected were mostly the main effects and there were only a few interactions. The ATTD of starter diets was affected by AME_N_ reduction, showing 3.7%-point higher organic matter digestibility in treatment Ross-0 compared to Ross-200. Organic matter ATTD and AME_N_ concentration were both unaffected by the expander prior to pelleting in the starter phase. However, EEh digestibility was improved when expander conditioning prior to pelleting was used (*p* = 0.046). In the grower phase, only the EEh was positively affected by expander conditioning prior to pelleting (*p* = 0.027). In the finisher phase, the AME_N_ concentration reduction decreased the organic matter.

The interactions of AME_N_ reduction vs. expander are shown in Table 7 for the AME_N_ concentration in the starter phase and EEh digestibility in the finisher phase. The measured AME_N_ concentration was numerically higher when the expander conditioning prior to pelleting was used. Considering the pelleted-only diet (without expander conditioning), the EEh digestibility was reduced, being lowest at the lowest AME_N_ concentration (Ross-200).

Results of starch and amino acids apparent ileal digestibility (AID) of the compound diets, as measured at the end of the performance trial, are summarized in Table 8. No interaction between the factors AME_N_ reduction and expander conditioning prior to pelleting was observed. In contrast, the main effect of AME_N_ concentration reduction was significant for a range of amino acids, whereas expander conditioning prior to pelleting poorly affected the AID of amino acids. Being lower as the AME_N_ concentration was reduced.

Detailed examination of AID values showed that treatment Ross-200 resulted in the highest starch digestibility, whereas TSAA, Thr, Arg, Leu, Phe, Gly, Ser, Pro, and Asp all had lower AID values when treatment Ross-160 was fed. Only the digestibility of TSAA (Table 8) and Pro were negatively affected by expander conditioning prior to pelleting.

Diet composition hardly changed the broiler performance (Table 9). Body weight gain was not affected by the main effects of AME_N_ concentration reduction or expander conditioning prior to pelleting. However, the factors interacted for BWG for the overall period (1–35 d). The treatment Ross-40 without expander conditioning prior to pelleting promoted the highest BWG (Table 10). In contrast, BWG was similar among the different AME_N_ concentration reductions (Ross-0 to Ross-200) when an expander prior to pelleting was used. The feed intake (FI) was not affected by treatments in grower, finisher, and overall feed phases. However, at the starter phase, the AME_N_ reduction interacted with expander conditioning prior to pelleting (Table 10). The highest FI was observed in treatment Ross-160 after expander conditioning prior to pelleting. Without expander conditioning prior to pelleting, the FI increased also with decreasing AME_N_ concentration in the diet. There were no differences in the comparison between expander conditioning within each AME_N_ concentration reduction.

The feed conversion ratio (FCR) was mostly influenced by treatment factors among performance parameters. Regarding main effects, the reduction in AME_N_ concentration affected FCR in the starter and finisher phases, and in the overall experimental period, getting worse as a lower AME_N_ concentration was calculated in the diet.

The FCR was reduced by 0.056 points for the overall period (Table 9). The expander conditioning prior to pelleting improved FCR in the overall period (1–35 d) regardless of the reduction in AME_N_ concentration. In the grower phase, the factors interacted. FCR was lowest when the combination of treatments Ross-120 and expander conditioning prior to pelleting were applied (Table 10). No differences in FCR were observed in the comparison between expander conditioning within each AME_N_ concentration reduction.

Internal organs and health indicators from blood serum were measured on day 36 of life. As shown in Table 11, just the liver weight, presented as a percentage of the non-fasted live body weight, was changed by the AME_N_ concentration reduction. Treatment Ross-200 increased the liver weight, relative to that of treatment Ross-40. For blood serum indicators, only the α-amylase activity showed higher values in broilers of treatment Ross-160 compared to Ross-0. Expander conditioning prior to pelleting did not influence these animal response parameters. No interaction was observed.

Commercial cuts were hardly changed by treatments (Table 12); although, total carcass yield was slightly higher when diets were just pelleted, and the AME_N_ concentration reduction resulted in lower abdominal fat deposition. There were no interactions observed for carcass yield, abdominal fat, and commercial cuts.

## 4. Discussion

One of the goals of the present study was to find out if energy coming from nutrients could be replaced by energy made available from non-nutritional sources, i.e., by the processing of compound feed with modifications at the processing line that could help to improve the pellet quality and animal response. As observed for the feed processing indicators (Table 4), the AME_N_ reduction is implied by the higher pressure applied by the pellet press to the compound feed. This resulted in higher consumption of electric power when diets had less AME_N_. The lower amount of soybean oil added to the diet may explain this. Oil greases the die holes, reducing friction, so the mechanical energy to push the compound feed through the die holes is lower, reflected in lower kWh/t, PDI, hardness, and higher content of fines (Figure 1). As explained by Abdollahi et al. [4] with increasing fat added to the compound feed, the starch particles become covered, restricting the steam penetration to the granules, and reducing starch gelatinization during the conditioning and later in the pellet die. In consequence, the friction is reduced, and agglomeration capacity is lowered, which weakens the resulting pellets, as found in this trial (Figure 1). On the one hand, although the AME_N_ concentration of the compound feed increased when fat was added, the higher fat content decreased pellet quality, as observed by decreasing PDI values (Figure 1). This result is supported by McKinney and Teeter [3]. However, fat is included to cover the animal requirements of fatty acids and energy, but sometimes these inclusions are high, challenging the pellet quality. On the other hand, expander conditioning prior to pelleting allows higher fat inclusion in the compound feed without substantial changes in the pellet quality [22], as can be observed in Table 5 and Figure 1. Our results showed that an average 4.7%-point lower PDI was achieved when 3.73% of soybean oil was included and diets were processed on the line without expander conditioning. However, with expander conditioning prior to pelleting, the decrease in PDI was only about 1.5% points. The results suggest that expander conditioning prior to pelleting enables the higher inclusion of fat in compound feed for broilers without losses in pellet quality, a goal for the feed mill.

Furthermore, it should be noted that the diet with the lowest PDI in this trial was the one without energy reduction (Ross-0) and pelleted without expander conditioning, as observed in Table 4. However, compound feed with a PDI of 89.7% and 6.3% of fines still represents a diet with well-performed processing, especially when animal responses are highlighted. McKinney and Teeter [3] showed that diets containing 60% of pellets and 40% fines had similar broiler performance to broilers fed with 100% pellets, supporting the absence of well-defined differences in feed intake and body weight gain, observed in the current trial. During feed processing in this trial, the aim was to produce pellets with a PDI as high as possible to demonstrate that higher digestibility and broiler performance with expander conditioning prior to pelleting is possible. However, PDI did not substantially affect animal performance in the present study, as the correlations with animal responses showed (Table 13, Table 14, Table 15, Table 16 and Table 17). This finding is supported by Svihus [23], who explained the high ability of broilers to digest corn starch, such as was observed in this trial. In fact, the diets had on average 449 g/kg of starch, being the highest source of energy, but the changes promoted by the expander conditioning prior to pelleting were not enough to improve starch digestibility. However, starch digestibility did not decrease with expander conditioning, showing that expander conditioning prior to pelleting contributes to producing high-quality pellets.

The effect of the expander conditioning prior to pelleting can be seen in the disruption of grain particles, resulting in the higher availability of nutrients for digestion. In fact, the EEh digestibility was higher in the starter and grower phases when expander conditioning prior to pelleting was applied, but the effect disappeared in the finisher phase. Younger birds have a reduced digestion capacity of the gastrointestinal tract, as described by Furlan and Macari [24]. On the one hand, the expander conditioning prior to pelleting may have increased the access of digestion enzymes to the lipid fraction of ingredients of the compound feed, improving fat digestibility. On the other hand, the experimental factors hardly changed the measured AME_N_, which could be explained by the similar results observed for the rearing of broilers in pens littered with wood shavings, which could have been eaten by the birds, affecting the excreta used to measure energy. These findings support the notion that expander conditioning prior to pelleting plays an important role in providing energy to the broilers by raising fat digestibility and likely enhancing starch digestibility.

The absence of substantial effects of AME_N_ reduction suggests that the energy concentration of diets may be reduced at feed formulation. Both processing lines, with or without an expander prior to pelleting, promoted high-quality pellets and showed few changes in pellet physical aspects as well as in digestibility. However, the energy requirements of modern broiler strains may support the reduction in AME_N_ concentration without substantial negative effects. Aftab [6] supports these findings by reviewing the energy and amino acid requirements of broilers. In that publication, the author suggests that the current strains require diets with lower AME_N_ and higher amino acid concentrations than recommended, especially due to the shorter time of approximately 35 days required to achieve slaughter weight. Therefore, the AME_N_ of diets for broilers can be reduced preserving the amino acid inclusion levels.

The apparent ileal amino acids digestibility of compound diets was also affected by treatments (Table 8). The diet without AME_N_ reduction was for most amino acids, the one with the higher digestibility compared to treatment Ross-160. The reduction in AME_N_ was responsible for lower amino acid digestibility. In contrast, the expander conditioning prior to pelleting did not affect AA digestibility (exceptions for TSAA and Pro) at similar amino acid compositions of diets. However, only TSAA and proline were negatively affected by expander conditioning prior to pelleting. This means that shear forces, heat, residence time, and moisture content, all features resulting from expander conditioning [25,26], poorly affected the digestion of amino acids in the present study. The AID of amino acids also supports the reduction in AME_N_ and the use of expander conditioning.

Considering the difficulties in using digestibility values of processed compound feeds for feed formulation, the gains by high-quality pellets produced by expander conditioning prior to pelleting in broiler performance are associated with the shorter time [27] and energy expenditure during ingestion of feed [28,29]. Broiler performance was thereby affected only slightly by experimental factors (Table 9). In fact, the feed intake was higher in the starter phase when diets had lower energy. Birds are likely to try to compensate for the lower energy with higher feed intake, as observed by Leeson et al. [30,31]. These authors demonstrated that broilers eat to compensate for the limiting nutrients in their diet, which is reflected in poor FCR in these feeding phases, as observed in our study.

The compensation of low energy in diets with higher feed intake was not clearly observed in the grower or finisher phases in the present study (*p* < 0.10). Aftab [6] suggests that modern broiler genetic lines may no longer regulate their feed intake by diet nutrient density, but instead by just physical distension of the gastrointestinal tract. This is reflected in our study by the lack of a statistical effect on feed intake and in body weight gain, although AME_N_ concentration was reduced. In addition, the abdominal fat accumulated proportionally to the lower AME_N_ concentration in the diet. The expander conditioning prior to pelleting did not affect these results (Table 12). Reducing the energy concentration in diets resulted in the AME_N_ to amino acid ratio being affected. Our results support the reduction in AME_N_ concentration by 80, 120, and 160 kcal/kg in starter, grower, and finisher feed phases, respectively, without negative effects on animal performance and the proportion of commercial cuts.

Furthermore, the use of expander conditioning prior to pelleting is favorable to optimize FCR, being 1.376 without and 1.352 g/g in the overall period when expander conditioning prior to pelleting was used. There was a tendency shown (*p* < 0.10) for improved FCR when an expander was not used in the starter phase; however, this changes as broilers get older, resulting in the benefit observed for the overall period. The higher FCR observed using an expander in the starter phase could be explained by the changes in the gastrointestinal tract that happened due to aging and due to adaptation to the diet, such as lower exogen pancreatic enzymes and bile production for this at up to 14-day-old birds. The older birds were later well-adapted [24].

Looking closer at the relationship between the physical pellet quality and the nutrient digestibility in the starter phase by correlation analysis (Table 13), the PDI had a positive correlation with FCR in the starter phase, suggesting that higher physical quality is important for birds up to 14 days of age, even if these pellets were crumbled to achieve an edible size that was ingestible by chickens of just a few days old. Later, in the grower and finisher feed phases these correlations disappear (Table 14 and Table 15). Although some correlations were observed, the PDI in this trial was higher than 89%, and fines were lower than 7%, showing therefore the high feed quality of all diets. Low fines amounts were also used by McKinney and Teeter [3], who did not find differences in FCR when birds were consuming diets with 0 to 80% of fines. Stocking density was not an experimental factor, but was low, promoting animal welfare. However, industrial production systems normally use higher housing densities, raising the importance of good pellets allowing fast feed intake, highlighting again the importance of producing pellets of high physical quality.

Animal physiological answers to the treatments were also measured, aiming to evaluate variables related to animal health (Table 11). The blood serum variables measured show that fat digestibility changed the lipase secretion positively, which is physiologically expected, and it is important to see that the levels of fat in diets may be measured in blood (Table 16). The serum concentration of α-amylase increased as AME_N_ concentration was reduced (Table 11). This can be associated with the starch content in the diet, which increased slightly as AME_N_ was reduced. However, the starch digestibility was only slightly affected by the treatments. On the one hand, soybean oil content reduction was the biggest driver for AME_N_ variation among treatments, increasing liver size as the AME_N_ concentration decreased (Table 11). On the other hand, cholesterol and triglyceride concentrations were not affected by treatments, showing that the health status was not changed by AME_N_ reduction and compound feed processing. Ivanovich et al. [32] conducted a study of changing AME_N_ and amino acid concentrations, and no changes in amylase and lipase were found. However, their study was conducted at up to 10 days of age, which represents a period with strong intestinal tract development and enzymatic adaptations. The longer time of eating diets with less energy may help to explain the differences observed in serum amylase. Thus, treatments hardly change serum markers, showing the metabolic adaptation to maintain body homeostasis.

Besides the correlation between physical properties and animal response, the correlation analyses between pellet quality variables and size of gastrointestinal organs and abdominal fat (Table 17) showed that abdominal fat is the variable that is most sensitive in relation to variations in pellet quality, AME_N_ concentration, and nutrient digestibility. Among them, the most powerful factor influencing abdominal fat deposition is the AME_N_ concentration (Table 12), especially when the AME_N_ to amino acid ratio is increasing. However, higher fat content (soybean oil added) reduces pellet quality. Together with high abdominal fat deposition, the poultry industry is avoiding both. Furthermore, the abdominal fat accumulation clearly shows that gradual AME_N_ reduction was achieved and how sensitive the body composition is to the energy content in the diet.

## 5. Conclusions

The use of diets with lower AME_N_ concentration, and with coarse corn particle sizes, conditioned with an expander prior to pelleting, is recommended for broilers. All physical aspects of the diets used here were comparable to each other, and probably higher than the feed industry practices, reflecting the experimental processing plant arrangement. Our trial showed the importance of feed processing as a tool to improve livestock efficiency. However, it requires adjustments in feed formulation to consider changes in the nutritive value due to processing. In addition, our trial showed that the addition of plant oil may be reduced in diets, bringing two important benefits at once: (a) reducing an expensive ingredient of the diet and (b) improving the pellet quality using adequate processing technology.

## Figures and Tables

**Table 1:** Ingredient and nutrient composition of the experimental diets used as starter, as is.

Item	Ross-0	Ross-40	Ross-80	Ross-120	Ross-160	Ross-200
Ingredients, %						
Corn	55.46	56.80	57.08	57.21	58.59	55.43
Soybean meal	36.19	36.21	36.18	36.07	36.05	36.82
Soybean oil	2.95	2.10	1.46	0.86	0.00	0.02
Wheat bran	0.50	0.00	0.40	1.00	0.50	3.00
Dicalcium phosphate	1.46	1.47	1.46	1.45	1.46	1.40
Limestone	0.63	0.63	0.64	0.65	0.63	0.67
Salt	0.38	0.38	0.38	0.38	0.38	0.38
DL-Methionine 99%	0.34	0.33	0.33	0.33	0.33	0.32
L-Lysine 54.6%	0.35	0.35	0.35	0.34	0.35	0.30
L-Threonine 98.5%	0.11	0.10	0.10	0.10	0.06	0.09
L-Isoleucine	0.02	0.02	0.01	0.01	0.01	0.00
L-Valine	0.08	0.08	0.08	0.07	0.07	0.05
Choline chloride 60%	0.07	0.07	0.07	0.07	0.07	0.06
Vitamin and mineral mix ^1^	1.00	1.00	1.00	1.00	1.00	1.00
Phytase ^2^	0.01	0.01	0.01	0.01	0.01	0.01
Coccidiostatic ^3^	0.05	0.05	0.05	0.05	0.05	0.05
Probiotic ^4^	0.10	0.10	0.10	0.10	0.10	0.10
TiO_2_	0.30	0.30	0.30	0.30	0.30	0.30
Calculated or analyzed (in brackets) nutrient composition, as is, g/kg unless noted ^5^						
AME_N_, kcal/kg	3000	2960	2920	2880	2840	2800
DM	(889.9)	(893.6)	(895.3)	(886.5)	(891.7)	(890.3)
Starch	(404.7)	(431.5)	(419.5)	(414.3)	(417.3)	(411.0)
EEh ^6^	(52.1)	(47.2)	(40.5)	(36.5)	(29.0)	(27.5)
CP	22.2 (22.5)	22.2 (22.2)	22.3 (22.8)	22.3 (22.5)	22.3 (22.4)	22.8 (23.4)
Ca	0.96 (0.97)	0.96 (0.98)	0.96 (0.98)	0.96 (0.96)	0.96 (0.97)	0.96 (0.98)
Non-phytate P	0.48	0.48	0.48	0.48	0.48	0.48
Total P	0.70 (0.71)	0.70 (0.71)	0.70 (0.72)	0.71 (0.70)	0.71 (0.72)	0.71 (0.72)
Na	0.16	0.16	0.16	0.16	0.16	0.16
Choline, mg/kg	1700	1700	1700	1700	1700	1700
Dig. Lys	1.24 (1.39)	1.24 (1.40)	1.24 (1.36)	1.24 (1.40)	1.24 (1.38)	1.24 (1.44)
Dig. TSAA	0.91 (0.96)	0.91 (0.97)	0.91 (0.93)	0.91 (0.94)	0.91 (0.94)	0.91 (0.99)
Dig. Thr	0.79 (0.91)	0.79 (0.91)	0.79 (0.89)	0.79 (0.90)	0.79 (0.90)	0.79 (0.94)
Dig. Trp	0.24	0.23	0.24	0.24	0.24	0.24
Dig. Arg	1.34 (1.46)	1.34 (1.46)	1.35 (1.45)	1.35 (1.48)	1.35 (1.47)	1.38 (1.57)
Dig. Val	0.98 (1.09)	0.98 (1.09)	0.98 (1.09)	0.98 (1.07)	0.98 (1.08)	0.98 (1.11)
Dig. Ile	0.84 (0.98)	0.84 (0.98)	0.84 (0.96)	0.84 (0.95)	0.84 (0.96)	0.85 (0.99)

^1^ Supplied per kilogram of diet: vitamin A, 10,000 IU; vitamin D3, 4000 IU; vitamin E, 20 IU; vitamin K3, 4 mg; thiamine, 3 mg; riboflavin, 7.5 mg; pyridoxine, 4.5 mg; cyanocobalamin, 0.0225 mg, pantothenic acid, 19.5 mg; niacin, 69 mg; folic acid, 0.195 mg; biotin, 0.012 mg; iron, 16.8 mg; zinc, 80 mg; manganese, 100 mg; copper, 12 mg; iodine, 1 mg; selenium, 0.25 mg. ^2^ Optiphos^®^ 2500 FTY, Huvepharma EOOD, Sofia, Bulgaria. ^3^ Sacox^®^, 120 g/kg, Huvepharma EOOD, Sofia, Bulgaria. ^4^ Ecobiol^®^ *Bacillus amyloliquefaciens* CECT 5940, min. 1 × 10^9^ CFU/g, Evonik Operations GmbH, Nutrition & Care—Animal Nutrition, Essen, Germany. ^5^ Analyzed values are an average of 8 replications. ^6^ Acid hydrolyzed ether extract.

**Table 2:** Ingredient and nutrient composition of the experimental diets used as grower, as is.

Item	Ross-0	Ross-40	Ross-80	Ross-120	Ross-160	Ross-200
Ingredients, %						
Corn	63.08	63.61	64.68	65.24	66.06	64.76
Soybean meal	29.12	29.28	28.99	29.15	29.08	29.50
Soybean oil	3.30	2.59	1.80	1.11	0.36	0.02
Wheat bran	0.00	0.00	0.00	0.00	0.00	1.30
Dicalcium phosphate	1.30	1.29	1.29	1.29	1.29	1.26
Limestone	0.56	0.59	0.59	0.57	0.58	0.61
Salt	0.38	0.38	0.38	0.38	0.38	0.38
DL-Methionine 99%	0.26	0.27	0.27	0.27	0.26	0.26
L-Lysine (Biolys) 54.6%	0.31	0.30	0.31	0.30	0.30	0.27
L-Threonine 98.5%	0.08	0.08	0.08	0.08	0.08	0.07
L-Isoleucine	0.02	0.02	0.02	0.02	0.02	0.01
L-Valine	0.05	0.05	0.05	0.05	0.05	0.03
Choline chloride 60%	0.08	0.08	0.08	0.08	0.08	0.07
Vitamin and mineral mix ^1^	1.00	1.00	1.00	1.00	1.00	1.00
Phytase ^2^	0.01	0.01	0.01	0.01	0.01	0.01
Coccidiostatic ^3^	0.05	0.05	0.05	0.05	0.05	0.05
Probiotic ^4^	0.10	0.10	0.10	0.10	0.10	0.10
TiO_2_	0.30	0.30	0.30	0.30	0.30	0.30
Calculated and analyzed (in brackets) nutrient composition, as is, g/kg unless noted ^5^						
AME_N_, kcal/kg	3100	3060	3020	2980	2940	2900
DM	(896.1)	(889.7)	(897.0)	(892.0)	(887.2)	(892.0)
Starch	(455.9)	(448.4)	(467.9)	(452.6)	(473.1)	(465.2)
EEh ^6^	(56.0)	(51.5)	(45.5)	(41.0)	(33.0)	(29.5)
CP	19.2 (19.5)	19.3 (19.7)	19.2 (19.4)	19.4 (19.2)	19.4 (19.4)	19.7 (19.8)
Ca	0.87 (0.88)	0.87 (0.86)	0.87 (0.88)	0.87 (0.88)	0.87 (0.87)	0.87 (0.88)
Non-phytate P	0.43	0.43	0.43	0.43	0.43	0.43
Total P	0.65 (0.67)	0.65 (0.66)	0.66 (0.65)	0.66 (0.68)	0.66 (0.68)	0.66 (0.67)
Na	0.16	0.16	0.16	0.16	0.16	0.16
Choline, mg/kg	1600	1600	1600	1600	1600	1600
Dig. Lys	1.05 (1.20)	1.05 (1.18)	1.05 (1.22)	1.05 (1.13)	1.05 (1.17)	1.05 (1.19)
Dig. TSAA	0.78 (0.82)	0.79 (0.84)	0.79 (0.82)	0.79 (0.82)	0.79 (0.80)	0.79 (0.85)
Dig. Thr	0.67 (0.79)	0.68 (0.78)	0.68 (0.81)	0.68 (0.82)	0.68 (0.77)	0.68 (0.79)
Dig. Trp	0.20	0.20	0.20	0.20	0.20	0.20
Dig. Arg	1.14 (1.25)	1.14 (1.24)	1.14 (1.29)	1.14 (1.29)	1.14 (1.24)	1.16 (1.28)
Dig. Val	0.84 (0.94)	0.84 (0.92)	0.84 (0.96)	0.84 (0.96)	0.84 (0.94)	0.84 (0.92)
Dig. Ile	0.72 (0.83)	0.73 (0.82)	0.73 (0.86)	0.73 (0.86)	0.73 (0.84)	0.73 (0.83)

^1^ Supplied per kilogram of diet: vitamin A, 10,000 IU; vitamin D3, 4000 IU; vitamin E, 20 IU; vitamin K3, 4 mg; thiamine, 3 mg; riboflavin, 7.5 mg; pyridoxine, 4.5 mg; cyanocobalamin, 0.0225 mg, pantothenic acid, 19.5 mg; niacin, 69 mg; folic acid, 0.195 mg; biotin, 0.012 mg; iron, 16.8 mg; zinc, 80 mg; manganese, 100 mg; copper, 12 mg; iodine, 1 mg; selenium, 0.25 mg. ^2^ Optiphos^®^ 2500 FTY, Huvepharma EOOD, Sofia, Bulgaria. ^3^ Sacox^®^, 120 g/kg, Huvepharma EOOD, Sofia, Bulgaria.^4^ Ecobiol^®^ *Bacillus amyloliquefaciens* CECT 5940, min. 1 × 10^9^ CFU/g, Evonik Operations GmbH, Nutrition & Care—Animal Nutrition, Essen, Germany. ^5^ Analyzed values are an average of 8 replications. ^6^Acid hydrolyzed ether extract.

**Table 3:** Ingredient and nutrient composition of the experimental diets used as finisher, as is.

Item	Ross-0	Ross-40	Ross-80	Ross-120	Ross-160	Ross-200
Ingredients, %						
Corn	62.01	62.87	63.23	64.03	64.74	65.86
Soybean meal	29.08	28.96	29.32	29.27	29.30	28.95
Soybean oil	4.95	4.20	3.53	2.79	2.07	1.28
Dicalcium phosphate	1.08	1.08	1.07	1.07	1.07	1.07
Limestone	0.52	0.53	0.53	0.53	0.51	0.52
Salt	0.38	0.38	0.38	0.38	0.38	0.38
DL-Methionine 99%	0.24	0.24	0.24	0.23	0.23	0.23
L-Lysine (Biolys) 54.6%	0.21	0.21	0.19	0.19	0.19	0.20
L-Threonine 98.5%	0.05	0.05	0.04	0.04	0.04	0.04
L-Valine	0.01	0.01	0.00	0.00	0.00	0.00
Choline chloride 60%	0.06	0.06	0.06	0.06	0.06	0.06
Vitamin and mineral mix ^1^	1.00	1.00	1.00	1.00	1.00	1.00
Phytase ^2^	0.01	0.01	0.01	0.01	0.01	0.01
Probiotic ^3^	0.10	0.10	0.10	0.10	0.10	0.10
TiO_2_	0.30	0.30	0.30	0.30	0.30	0.30
Calculated and analyzed (in brackets) nutrient composition, as is, g/kg unless noted ^4^						
AME_N_, kcal/kg	3200	3160	3120	3080	3040	3000
DM	(885.7)	(896.2)	(891.1)	(896.5)	(890.0)	(888.5)
Starch	(437.0)	(450.4)	(439.7)	(452.3)	(450.1)	(463.5)
EEh ^5^	(72.5)	(71.5)	(61.0)	(54.0)	(50.0)	(41.5)
CP	18.9 (19.1)	18.9 (18.6)	19.1 (18.8)	19.1 (18.9)	19.2 (19.3)	19.1 (19.6)
Ca	0.79 (0.80)	0.79 (0.81)	0.79 (0.79)	0.79 (0.80)	0.79 (0.78)	0.79 (0.80)
Non-phytate P	0.39	0.39	0.39	0.39	0.39	0.39
Total P	0.61 (0.62)	0.61 (0.60)	0.61 (0.61)	0.62 (0.63)	0.62 (0.64)	0.62 (0.63)
Na	0.16	0.16	0.16	0.16	0.16	0.16
Choline, mg/kg	1500	1500	1500	1500	1500	1500
Dig. Lys	0.99 (1.14)	0.99 (1.10)	0.99 (1.10)	0.99 (1.10)	0.99 (1.13)	0.99 (1.11)
Dig. TSAA	0.75 (0.78)	0.75 (0.78)	0.75 (0.80)	0.75 (0.79)	0.75 (0.81)	0.75 (0.80)
Dig. Thr	0.64 (0.75)	0.64 (0.73)	0.64 (0.74)	0.64 (0.74)	0.64 (0.76)	0.64 (0.74)
Dig. Trp	0.20	0.20	0.20	0.20	0.20	0.20
Dig. Arg	1.13 (1.24)	1.13 (1.19)	1.14 (1.20)	1.14 (1.22)	1.14 (1.25)	1.14 (1.24)
Dig. Val	0.79 (0.89)	0.79 (0.87)	0.79 (0.87)	0.79 (0.87)	0.79 (0.90)	0.79 (0.88)
Dig. Ile	0.71 (0.81)	0.71 (0.79)	0.71 (0.81)	0.71 (0.78)	0.72 (0.82)	0.71 (0.80)

^1^ Supplied per kilogram of diet: vitamin A, 10,000 IU; vitamin D3, 4000 IU; vitamin E, 20 IU; vitamin K3, 4 mg; thiamine, 3 mg; riboflavin, 7.5 mg; pyridoxine, 4.5 mg; cyanocobalamin, 0.0225 mg, pantothenic acid, 19.5 mg; niacin, 69 mg; folic acid, 0.195 mg; biotin, 0.012 mg; iron, 16.8 mg; zinc, 80 mg; manganese, 100 mg; copper, 12 mg; iodine, 1 mg; selenium, 0.25 mg. ^2^ Optiphos^®^ 2500 FTY, Huvepharma EOOD, Sofia, Bulgaria. ^3^ Ecobiol^®^ *Bacillus amyloliquefaciens* CECT 5940, min. 1 × 10^9^ CFU/g, Evonik Operations GmbH, Nutrition & Care—Animal Nutrition, Essen, Germany. ^4^ Analyzed values are an average of 8 replications. ^5^ Acid hydrolyzed ether extract.

**Table 4:** Feed processing applied to diets and the pellet quality of diets.

Item	Steam, kg/t ^1^	P. Press, bar ^3^	SME, kWh/t ^2^	SME, kWh/t ^3^	PDI, %	Fines, % ^4^	Hardness, N
AME_N_							
Ross-0	59.0	28.2 ^c^		11.2 ^c^	92.1	5.73	33.2 ^d^
Ross-40	58.8	32.3 ^bc^		12.3 ^bc^	92.5	5.83	34.5 ^cd^
Ross-80	59.2	34.8 ^ab^		12.9 ^ab^	93.8	5.12	38.3 ^bcd^
Ross-120	58.2	36.2 ^ab^		13.6 ^ab^	94.6	4.80	43.4 ^abc^
Ross-160	58.1	36.7 ^a^		14.0 ^a^	94.9	4.37	49.4 ^a^
Ross-200	57.7	36.5 ^ab^		14.2 ^a^	94.6	4.80	47.5 ^ab^
Expander (Ep)							
With	60.3	30.9	9.40	11.7	95.5	4.27	42.3
Without	56.6	37.3	2.40	14.4	92.0	5.94	39.8
SEM	0.361	0.827	0.592	0.322	0.387	0.255	1.780
*p*-value							
AME_N_	0.105	<0.001	0.884	<0.001	<0.001	0.398	<0.001
Expander	<0.001	<0.001	<0.001	<0.001	<0.001	0.001	0.168
AME_N_ × Ep	0.363	0.945	0.884	0.871	0.009	0.963	0.350

^a > b > c > d^LSmeans with different superscripts in the same column are significantly different by Tukey–Kramer test, *p* < 0.05. ¹ Pre-conditioner temperature ~85 °C; ^2^ Expander OE 15, heat temperature ~106 °C, ^3^ Pellet press 38–780, die: 3 mm 1/8, 2 t/h, throughput; ^4^ <2.8 mm.

**Table 5:** Breakdown of significant interactions as presented in Table 4.

Item		Steam, kg/t ^1^	P. Press, bar ^3^	SME, kWh/t ^2^	SME, kWh/t ^3^	PDI, %	Fines, % ^4^	Hardness, N
AME_N_	Expander							
Ross-0		61.0	24.7	9.33	10.0	94.4 ^aA^	5.20	33.2
Ross-40		61.0	28.7	9.43	11.2	95.0 ^aA^	4.60	37.5
Ross-80	With	56.8	31.3	9.43	11.7	94.5 ^aA^	4.23	42.4
Ross-120		60.0	33.7	9.43	12.1	95.7 ^aA^	4.10	42.2
Ross-160		59.5	33.3	9.33	12.3	96.2 ^aA^	3.63	48.2
Ross-200		56.3	33.7	9.43	13.0	96.0 ^aA^	3.87	50.5
Ross-0		57.0	31.7		12.5	89.7 ^bB^	6.27	33.3
Ross-40		56.7	36.0		13.5	90.0 ^bB^	7.07	31.6
Ross-80	Without	56.8	38.3		14.2	92.1 ^bA^	6.00	34.2
Ross-120		56.5	38.7		15.1	93.6 ^aA^	5.50	44.7
Ross-160		56.8	40.0		15.8	93.6 ^bA^	5.10	50.6
Ross-200		56.3	39.3		15.4	93.2 ^bA^	5.73	44.5

^a > b^ Comparison between With and Without Expander, within AME_N_. ^A > B^ Comparison between AME_N_, within Expander. ¹ Pre-conditioner temperature ~85 °C; ^2^ Expander OE 15, heat temperature ~106 °C, ^3^ Pellet press type 38–780, die: 3 mm 1/8. N: Kahl hardness test method; ^4^ <2.8 mm.

**Table 6:** Broiler apparent total tract digestibility (%) and AME_N_ concentration, kcal/kg.

Item	Starter			Grower			Finisher		
	OM ^1^	Eeh ^2^	AME_N_	OM	EEh	AME_N_	OM	EEh	AME_N_
AME_N_									
Ross-0	75.1 ^a^	81.6	3451	78.0	84.9	3552	75.8 ^ab^	87.4	3589
Ross-40	74.3 ^ab^	81.3	3388	77.2	84.4	3514	76.8 ^a^	86.8	3625
Ross-80	74.0 ^ab^	82.0	3428	77.2	84.4	3555	76.0 ^ab^	86.9	3626
Ross-120	73.5 ^ab^	80.2	3413	77.5	85.0	3613	74.6 ^b^	86.1	3576
Ross-160	74.2 ^ab^	82.3	3338	77.2	85.2	3519	75.1 ^ab^	88.7	3539
Ross-200	71.4 ^b^	82.1	3303	76.3	86.1	3520	76.8 ^a^	86.8	3643
Expander (Ep)									
With	74.1	82.3	3406	77.5	85.8	3556	75.8	87.3	3605
Without	73.5	80.9	3368	77.0	84.2	3535	75.9	86.9	3594
SEM	0.350	0.599	13.835	0.166	0.571	11.890	0.197	0.418	14.615
*p*-value									
AME_N_	0.041	0.567	0.005	0.050	0.743	0.158	0.002	0.013	0.330
Expander	0.369	0.046	0.110	0.054	0.027	0.395	0.609	0.392	0.697
AME_N_ × Ep	0.211	0.435	0.021	0.238	0.372	0.468	0.348	0.021	0.278

^a > b^ LSmeans in column, superscripted with different letters differ significantly by Tukey–Kramer test, *p* < 0.05. ^1^ OM = organic matter. ^2^ EEh = acid hydrolyzed ether extract.

**Table 7:** Breakdown of significant interactions as presented in Table 6.

Item		Starter AME_N_	Finisher EEh, %
AME_N_	Expander		
Ross-0	With	3446 ^abA^	86.2 ^aA^
Ross-40		3357 ^abA^	86.5 ^aA^
Ross-80		3540 ^aA^	87.3 ^aA^
Ross-120		3421 ^abA^	87.3 ^aA^
Ross-160		3373 ^bA^	89.1 ^aA^
Ross-200		3301 ^bA^	87.3 ^aA^
Ross-0	Without	3457 ^aA^	88.6 ^aA^
Ross-40		3304 ^aA^	88.2 ^aA^
Ross-80		3304 ^aA^	86.2 ^abA^
Ross-120		3419 ^aA^	87.1 ^abA^
Ross-160		3316 ^aB^	86.6 ^abA^
Ross-200		3404 ^aA^	84.8 ^bA^

^a > b^ Comparison between AME_N_ within Expander. ^A > B^ Comparison between Expander within AME_N_.

**Table 8:** Broiler apparent ileal digestibility of starch and amino acids, %.

Item	Starch	Met	Cys	TSAA	Lys	Thr	Arg	Ile	Leu	Val	His	Phe	Gly	Ser	Pro	Ala	Asp
AME_N_																	
Ross-0	91.7 ^ab^	93.6	79.2	88.6 ^a^	90.3	82.3 ^a^	92.5 ^a^	85.9	87.6 ^a^	86.6	88.0	88.7 ^a^	83.5 ^a^	85.9 ^a^	86.6 ^a^	85.7	86.1 ^a^
Ross-40	92.3 ^ab^	93.1	77.9	87.8 ^ab^	89.6	81.2 ^ab^	92.2 ^a^	84.9	86.8 ^ab^	85.7	87.0	88.0 ^ab^	82.6 ^a^	84.7 ^ab^	85.3 ^ab^	84.6	85.6 ^ab^
Ross-80	92.1 ^ab^	93.4	78.0	88.0 ^ab^	89.7	81.4 ^ab^	92.2 ^a^	85.3	87.2 ^ab^	85.9	87.2	88.0 ^ab^	82.7 ^a^	85.1 ^ab^	85.6 ^a^	85.0	85.6 ^ab^
Ross-120	91.1 ^b^	93.0	77.6	87.6 ^ab^	89.4	80.8 ^ab^	91.9 ^ab^	84.6	86.4 ^ab^	85.1	86.5	87.4 ^ab^	82.0 ^ab^	84.4 ^ab^	84.7 ^ab^	83.9	85.1 ^ab^
Ross-160	91.6 ^ab^	91.6	74.0	85.5 ^b^	87.6	78.3 ^b^	90.3 ^b^	81.9	84.6 ^b^	83.0	84.2	85.4 ^b^	79.6 ^b^	82.0 ^b^	82.9 ^b^	81.6	82.9 ^b^
Ross-200	92.7 ^a^	92.5	76.1	87.3 ^ab^	89.2	81.4 ^ab^	91.7 ^ab^	83.0	87.1 ^ab^	86.3	85.5	87.4 ^ab^	82.1 ^ab^	84.1 ^ab^	85.3 ^ab^	82.8	84.8 ^ab^
Expander (Ep)																	
With	92.2	92.5	76.2	86.9	88.9	80.5	91.5	83.8	86.3	84.9	85.9	87.0	81.7	83.8	84.6	83.3	84.6
Without	91.6	93.3	78.1	88.0	89.7	81.3	92.1	84.7	87.0	85.9	86.9	87.9	82.5	84.9	85.6	84.5	85.5
SEM	0.162	0.302	0.622	0.287	0.259	0.381	0.188	0.623	0.312	0.317	0.545	0.285	0.303	0.336	0.284	0.685	0.314
*p*-value																	
AME_N_	0.048	0.382	0.197	0.027	0.050	0.025	0.010	0.434	0.035	0.075	0.367	0.017	0.003	0.020	0.002	0.520	0.038
Expander	0.063	0.170	0.125	0.039	0.096	0.231	0.118	0.458	0.252	0.195	0.354	0.092	0.106	0.085	0.049	0.349	0.142
AME_N_ × Ep	0.618	0.952	0.683	0.665	0.688	0.128	0.759	0.833	0.080	0.079	0.835	0.793	0.427	0.628	0.207	0.876	0.748

^a > b^LSmeans in column, superscripted with different letters differ significantly by Tukey–Kramer test, *p* < 0.05.

**Table 9:** Broiler performance from 1 to 35 days.

Item	BWG, g				FI, g				FCR, g:g			
	1–14 d	15–28 d	29–35 d	1–35 d	1–14 d	15–28 d	29–35 d	1–35 d	1–14 d	15–28 d	29–35 d	1–35 d
AME_N_												
Ross-0	493.8	1330	880.1	2704	548.6	1841	1244	3631	1.111 ^b^	1.384	1.413 ^b^	1.343 ^b^
Ross-40	502.2	1327	935.1	2764	564.5	1831	1319	3720	1.124 ^b^	1.380	1.411 ^b^	1.346 ^ab^
Ross-80	499.5	1341	896.7	2737	564.4	1865	1302	3752	1.130 ^b^	1.391	1.452 ^ab^	1.371 ^ab^
Ross-120	490.6	1355	889.0	2734	567.1	1826	1340	3707	1.156 ^a^	1.348	1.491 ^ab^	1.356 ^ab^
Ross-160	492.7	1325	883.4	2701	577.9	1877	1281	3698	1.173 ^a^	1.417	1.450 ^ab^	1.369 ^ab^
Ross-200	489.0	1356	889.7	2734	573.1	1892	1375	3825	1.172 ^a^	1.395	1.546 ^a^	1.399 ^a^
Expander (Ep)												
With	492.2	1341	888.7	2721	565.5	1846	1280	3677	1.149	1.377	1.440	1.352
Without	497.1	1337	902.6	2737	566.7	1865	1337	3766	1.140	1.395	1.481	1.376
SEM	2.183	7.853	7.605	13.320	2.386	7.895	9.156	16.900	0.004	0.007	0.013	0.006
*p*-value												
AME_N_	0.129	0.590	0.136	0.254	0.001	0.582	0.139	0.078	<0.001	0.018	0.048	0.050
Expander	0.129	0.800	0.267	0.355	0.442	0.860	0.274	0.583	0.088	0.111	0.123	0.031
AME_N_ × Ep	0.076	0.110	0.539	0.024	0.024	0.285	0.849	0.271	0.810	0.001	0.906	0.246

^a > b^ LSmeans in column, superscripted with different letters differ significantly by Tukey–Kramer test, *p* < 0.05.

**Table 10:** Breakdown of significant interactions as presented in Table 9.

Item		BWG, 1–35 d	FI, 1–14 d	FCR, 15–28 d
AME_N_	Expander			
Ross-0	With	2674 ^aA^	559.5 ^bA^	1.409 ^aA^
Ross-40		2697 ^aA^	556.5 ^bA^	1.399 ^aA^
Ross-80		2741 ^aA^	562.9 ^bA^	1.397 ^aA^
Ross-120		2745 ^aA^	577.6 ^abA^	1.295 ^bA^
Ross-160		2719 ^aA^	592.9 ^aA^	1.403 ^aA^
Ross-200		2745 ^aA^	584.5 ^abA^	1.360 ^abA^
Ross-0	Without	2734 ^abA^	561.4 ^bA^	1.359 ^aA^
Ross-40		2831 ^aA^	577.5 ^abA^	1.361 ^aA^
Ross-80		2733 ^abA^	581.4 ^aA^	1.386 ^aA^
Ross-120		2723 ^abA^	574.3 ^aA^	1.401 ^aA^
Ross-160		2683 ^bA^	573.0 ^aA^	1.431 ^aA^
Ross-200		2717 ^abA^	584.6 ^aA^	1.429 ^aA^

^a > b^ Comparison between AME_N_ within Expander; ^A > B^ Comparison between Expander within AME_N_.

**Table 11:** Broiler internal organs, blood serum health indicators from broilers slaughtered at 36 days, % of body weight, or as noted.

Item	BW ^1^, g	Heart	Giz. *	Giz., pH	Pancreas	Liver	Protein ^2^	Cholesterol ^2^	Triglyceride ^2^	GGT ^2^	Lipase ^2^	α-Amylase ^2^
AME_N_												
Ross-0	2881	0.64	1.24	2.77	0.19	2.18 ^ab^	3.08	138.2	86.3	20.8	15.5	354.1 ^b^
Ross-40	2936	0.64	1.23	2.80	0.20	2.11 ^b^	3.22	133.8	85.5	19.3	18.4	355.8 ^ab^
Ross-80	2876	0.63	1.29	2.89	0.21	2.18 ^ab^	3.18	136.9	91.4	19.1	19.5	428.3 ^ab^
Ross-120	2906	0.63	1.21	2.99	0.21	2.14 ^ab^	3.40	144.5	87.1	18.5	17.4	408.8 ^ab^
Ross-160	2888	0.61	1.26	3.05	0.20	2.18 ^ab^	3.25	141.7	91.2	19.4	18.9	454.5 ^a^
Ross-200	2901	0.60	1.25	2.93	0.20	2.27 ^a^	3.35	139.2	91.1	19.7	19.0	425.9 ^ab^
Expander (Ep)												
With	2899	0.63	1.24	2.88	0.21	2.17	3.21	139.4	85.3	19.2	18.6	418.7
Without	2897	0.63	1.26	2.93	0.20	2.18	3.29	138.6	92.2	17.8	17.6	390.5
SEM	15.081	0.007	0.022	0.051	0.003	0.015	0.039	1.525	2.666	0.279	1.138	11.861
*p*-value												
AME_N_	0.622	0.187	0.562	0.133	0.601	0.037	0.130	0.356	0.967	0.131	0.893	0.047
Expander	0.910	0.978	0.515	0.433	0.290	0.578	0.226	0.782	0.187	0.252	0.623	0.197
AME_N_ × Ep	0.209	0.918	0.780	0.158	0.680	0.816	0.398	0.207	0.440	0.887	0.934	0.787

^a > b^ LSmeans in column, superscripted with different letters differ significantly by Tukey–Kramer test, *p* < 0.05. ^1^ Non-fasted. ^2^ Protein, g/dL; cholesterol, mg/dL; triglyceride, mg/dL; GGT (gamma-glutamyl transferase), U/L; Lipase, U/L; α-Amylase, U/L. * Gizzard.

**Table 12:** Slaughter performance of broilers and abdominal fat, %.

Item	Carcass ^1^	Abd Fat ^2^	Breast Fillet ^2^	Breast Tenders ^2^	Legs ^2^	Wings ^2^
AME_N_						
Ross-0	73.1	1.81 ^a^	6.30	29.2	28.9	9.89
Ross-40	73.2	1.76 ^ab^	6.36	29.4	28.8	9.92
Ross-80	73.4	1.65 ^ab^	6.33	28.8	28.7	9.81
Ross-120	73.4	1.56 ^b^	6.41	29.2	28.9	10.0
Ross-160	73.1	1.57^b^	6.26	28.7	29.1	9.94
Ross-200	73.7	1.49^b^	6.49	28.9	29.0	10.0
Expander (Ep)						
With	73.2	1.61	6.41	29.1	28.8	10.0
Without	73.5	1.66	6.31	29.0	29.0	9.90
SEM	0.069	0.193	0.397	1.147	0.983	0.353
*p*-value						
AME_N_	0.058	0.001	0.769	0.572	0.974	0.597
Expander	0.022	0.267	0.293	0.629	0.702	0.356
AME_N_ × Ep	0.398	0.291	0.361	0.822	0.587	0.111

^a > b^ LSmeans in column, superscripted with different letters differ significantly by Tukey–Kramer test, *p* < 0.05. ^1^ Relative to live body weight. ^2^ Relative to carcass weight.

**Table 13:** Pearson’s correlations between feed physical proprieties and digestible and performance values of broilers in starter feed phase, 1 to 14 d.

Item	Fat, dig.	OM, dig.	AME_N_	BWG, g	FI, g	FCR, g:g
PDI	−0.126	−0.001	0.001	−0.214	0.081	0.405
*p*-value	0.2904	0.99	0.99	0.0716	0.50	0.0004
N	0.145	−0.014	−0.203	−0.124	0.251	0.481
*p*-value	0.22	0.91	0.0871	0.29	0.0358	<0.0001
Fines	0.042	−0.077	0.003	0.150	−0.052	−0.306
*p*-value	0.72	0.52	0.52	0.21	0.67	0.0089

**Table 14:** Pearson’s correlations between feed physical proprieties and digestible and performance values of broilers in grower feed phase, 15 to 28 d.

Item	Fat, dig.	OM, dig.	AME_N_	BWG, g	FI, g	FCR, g:g
PDI	−0.088	−0.042	0.081	−0.041	0.018	0.099
*p*-value	0.45	0.72	0.49	0.73	0.88	0.41
N	−0.005	−0.142	0.020	0.082	0.041	0.029
*p*-value	0.96	0.23	0.86	0.49	0.73	0.81
Fines	0.049	0.014	−0.093	0.012	0.113	0.030
*p*-value	0.68	0.90	0.43	0.91	0.34	0.80

**Table 15:** Pearson’s correlations between feed physical proprieties and digestible and performance values of broilers in finisher feed phase, 29 to 35 d.

Item	Fat, dig.	OM, dig.	AME	BWG, g	FI, g	FCR, g:g
PDI	−0.004	−0.166	−0.087	−0.187	0.095	−0.020
*p*-value	0.96	0.16	0.46	0.11	0.42	0.87
N	0.027	−0.146	−0.053	−0.179	0.104	0.231
*p*-value	0.81	0.22	0.65	0.13	0.38	0.0684
Fines	−0.049	−0.103	−0.051	−0.051	−0.181	0.050
*p*-value	0.68	0.38	0.66	0.66	0.12	0.69

**Table 16:** Pearson’s correlations between feed physical proprieties, performance, and blood serum variables of broilers slaughtered at 36 days of age.

Item	Protein, g/dL	Cholesterol, mg/dL	GGT, U/L	Lipase, U/L	α-Amylase, U/L	Triglyceride, mg/dL
PDI	0.060	0.126	−0.186	0.104	0.290	−0.039
*p*-value	0.61	0.29	0.11	0.38	0.0138	0.74
N	0.252	0.163	−0.141	0.085	0.238	0.081
*p*-value	0.0326	0.17	0.23	0.47	0.0444	0.49
Fines	−0.001	−0.189	0.066	−0.012	−0.087	0.137
*p*-value	0.99	0.11	0.58	0.92	0.92	0.24
Fat dig	−0.074	−0.299	−0.274	0.375	−0.267	−0.203
*p*-value	0.53	0.0108	0.0199	0.0012	0.0238	0.0863
OM dig	−0.235	−0.080	0.075	0.149	−0.076	−0.240
*p*-value	0.0465	0.50	0.53	0.21	0.52	0.0418
AME_N_	−0.284	−0.022	0.118	0.021	0.063	−0.141
*p*-value	0.0157	0.85	0.32	0.85	0.59	0.34
BWG, 29–35d	0.070	−0.195	−0.327	0.295	−0.319	0.143
*p*-value	0.55	0.0995	0.0050	0.0119	0.0063	0.23
FI 29–35d	0.183	−0.105	−0.300	0.447	−0.252	0.027
*p*-value	0.12	0.38	0.0104	<0.0001	0.0327	0.82
FCR, 29–35d	0.360	−0.018	0.041	0.048	0.089	0.078
*p*-value	0.0038	0.88	0.75	0.70	0.48	0.54

**Table 17:** Pearson’s correlations between feed physical proprieties and internal organs weight relative to live body weight and gizzard content pH and abdominal fat of broilers slaughtered at 36 and 37 days of age, respectively.

Item	Gizzard Content, pH	Heart, g	Liver, g	Pancreas, g	Gizzard, g	Abd. Fat ^1^
PDI	0.067	−0.088	0.055	0.162	−0.031	−0.313
*p*-value	0.55	0.46	0.64	0.17	0.79	0.0073
N	0.149	−0.16	0.181	0.092	−0.047	−0.389
*p*-value	0.20	0.17	0.12	0.43	0.69	0.0008
Fines	−0.027	0.055	0.036	−0.061	0.028	0.172
*p*-value	0.82	0.64	0.76	0.61	0.81	0.14
Fat dig	0.537	−0.404	−0.283	−0.481	0.663	0.328
*p*-value	<0.0001	0.0004	0.0158	<0.0001	<0.0001	0.0049
OM dig	−0.002	−0.070	−0.090	−0.155	0.062	0.162
*p*-value	0.98	0.55	0.44	0.19	0.60	0.17
AME_N_	−0.012	−0.103	0.042	−0.013	0.010	0.084
*p*-value	0.91	0.38	0.72	0.91	0.93	0.48

^1^ Abdominal fat weight relative to carcass weight.

## Data Availability

No new data were created or analyzed in this study. Data sharing is not applicable to this article.

## References

[1] Ahmad H., Khalique A., Naveed S., Zia M.W., Zahid U., Moeed A. (2017). Efficacy of a synthetic antioxidant treatment in stabilizing poultry byproduct meal and subsequent impact of the treated meal on selected growth parameters of broilers. Braz. J. Poult Sci..

[2] Lee B.B., Yang T.S., Goo D., Choi H.S., Pitargue F.M., Jung H., Kil D.Y. (2018). Effects of dietary β-mannanase supplementation on the additivity of true metabolizable energy values for broiler diets. Asian-Australas J. Anim. Sci..

[3] McKinney L.J., Teeter R.G. (2004). Predicting effective value of nonnutritive factors: I. Pellet quality and II. Prediction of consequential formulation dead zones. Poult. Sci..

[4] Abdollahi M.R., Ravindran V., Svihus B. (2013). Pelleting of broiler diets: An overview with emphasis on pellet quality and nutritional value. Anim. Feed Sci. Technol..

[5] Lundblad K.K., Issa S., Hancock J.D., Behnke K.C., McKinney L.J., Alavi S., Prestøkken E., Fledderus J., Sørensen M. (2011). Effects of steam conditioning at low and high temperature, expander conditioning and extruder processing prior to pelleting on growth performance and nutrient digestibility in nursery pigs and broiler chickens. Anim. Feed Sci. Technol..

[6] Aftab U. (2019). Energy and amino acid requirements of broiler chickens: Keeping pace with the genetic progress. World Poult. Sci. Assoc..

[7] Maharjan P., Mullenix G., Hilton K., Caldas J., Beitia A., Weil J., Coon C. (2020). Effect of digestible amino acids to energy ratios on performance and yield of two broiler lines housed in different grow-out environmental temperatures. Poult. Sci..

[8] Boroojeni F.G., Svihus B., von Reichenbach H.G., Zentek J. (2016). The effects of hydrothermal processing on feed hygiene, nutrient availability, intestinal microbiota and morphology in poultry—A review. Anim. Feed Sci. Technol..

[9] Liu S.Y., Selle P.H. (2015). A consideration of starch and protein digestive dynamics in chicken-meat production. World. Poult. Sci. Assoc..

[10] Puntigam R., Schedle K., Schwarz C., Wanzenböck E., Eipper J., Lechner E.-M., Yin L., Gierus M. (2017). Very high expander processing of maize on animal performance, digestibility and product quality of finishing pigs and broilers. Animal.

[11] AminoDat® 5.0 (2016). Animal Nutritionist’s Information Edge.

[12] Aviagen (2014). Ross Nutritional Specifications. http://garantitavukculuk.com/doc/Ross_nutrition_spec.pdf.

[13] Pfost H.B. (1963). Testing the durability of pelleted feed. Feedstuffs.

[14] Naumann C., Bassler C. (2012). Die Chemische Untersuchung von Futtermitteln.

[15] AOAC (2006). Official Methods of Analysis.

[16] Figueiredo-Silva C., Lemme A., Sangsue D., Kiriratnikom S. (2015). Effect of DL-methionine supplementation on the success of almost total replacement of fish meal with soybean meal in diets for hybrid tilapia (*Oreochromis niloticus* × *Oreochromis mos-sambicus*). Aquac. Nutr..

[17] Jagger S., Wiseman J., Cole D.J.A., Craigon J. (1992). Evaluation of inert markers for the determination of ileal and faecal apparent digestibility values in the pig. Br. J. Nutr..

[18] Kong C., Adeola O. (2014). Evaluation of amino acid and energy utilization in feedstuff for swine and poultry diets. Asian Australas. J. Anim. Sci..

[19] Shapiro S. (1965). An analysis of variance test for normality (complete samples). Biometrika.

[20] SAS (2009). User’s Guide.

[21] Tukey J. (1991). The philosophy of multiple comparison. Stat. Sci..

[22] Fancher B.I., Rollins D., Trimbee B. (1996). Feed processing using the annular gap expander and its impact on poultry performance. J. Appl. Poult. Res..

[23] Svihus B. (2014). Starch digestion capacity of poultry. Poult. Sci..

[24] Furlan R.L., Macari M. (2002). Lipídios: Digestão e absorção. Fisiologia Aviária Aplicada a Frangos de Corte.

[25] Thomas M., van Vliet T., van der Poel A.F.B. (1998). Physical quality of pelleted animal feed: 3 Contribution of feedstuff components. Anim. Feed Sci. Technol..

[26] Voragen A.G.J., Gruppen H., Marsman G.J.P., Mul A.J., Garnsworthy P.C., Cole D.J.A. (1995). Effect of some manufacturing technologies on chemical, physical and nutritional properties of feed. Recent Advances in Animal Nutrition.

[27] Jensen L.S., Merrill L.H., Reddy C.V., McGinnis J. (1962). Observations on eating patterns and rate of food passage of birds fed pelleted and unpelleted diets. Poult. Sci..

[28] Engberg R.M., Hedemann M.S., Jensen B.B. (2002). The influence of grinding and pelleting of feed on the microbial composition and activity in the digestive tract of broiler chickens. Br. Poult. Sci..

[29] Svihus B., Kløvstad K.H., Perez V., Zimonja O., Sahlstrom S., Schuller R.B., Jeksrud W.K., Prestløkken E. (2004). Physical and nutritional effects of pelleting of broiler chicken diets made from wheat ground to different coarsenesses by the use of roller mill and hammer mill. Anim. Feed Sci. Technol..

[30] Leeson S., Caston L., Summers J.D. (1996). Broiler response to diet energy. Poult. Sci..

[31] Leeson S., Caston L., Summers J.D. (1996). Broiler response to energy or energy and protein dilution in finisher phase. Poult. Sci..

[32] Ivanovich F.V., Karlovich A.O., Mahdavi R., Afanasyevich E.I. (2017). Nutrient density of prestarter diets from 1 to 10 days of age affects intestinal morphometry, enzyme activity, serum indices and performance of broiler chickens. Anim. Nutr..

